# Experimental and Theoretical Study of the Synthesis of a Deep Eutectic Solvent Based on Protonated Caffeine, Ethylene Glycol, and ZnCl_2_

**DOI:** 10.3390/molecules30071557

**Published:** 2025-03-31

**Authors:** Laura Sofía Benavides-Maya, Manuel Felipe Torres-Perdomo, Luz M. Ocampo-Carmona, Luver Echeverry-Vargas

**Affiliations:** 1 Departamento de Materiales y Minerales, Universidad Nacional de Colombia, Medellin 050034, Colombia; lsbenavidesm@unal.edu.co (L.S.B.-M.); mftorrespe@unal.edu.co (M.F.T.-P.);; 2 Department of Metallurgical Engineering, University of Concepcion, Concepción 4070371, Chile

**Keywords:** deep eutectic solvents (DESs), protonated caffeine (CafCl), ab initio molecular dynamics (AIMD)

## Abstract

In this study, a deep eutectic solvent (DES) incorporating protonated caffeine (CafCl), ethylene glycol (EG), and zinc chloride (${\mathrm{ZnCl}}_{2}$) was synthesized and characterized for the first time. Caffeine was protonated using an optimized procedure in an anhydrous medium to enhance its interaction with the system, and its structure was confirmed by FTIR spectroscopy, NMR, and thermogravimetric analysis (TGA), evidencing the formation of the N-H bond in the imidazole ring. A eutectic mixture with a molar ratio of ${\mathrm{ETG:ZnCl}}_{2}$:CafCl of 1:2:0.1 was synthesized, and its characterization confirmed the formation of hydrogen bonds and the coordinative interaction between the components. Additionally, computational simulations based on COSMO-RS and ab initio molecular dynamics (AIMD) were conducted to analyze the charge distribution and the stability of the hydrogen bond network in the eutectic mixture. Sigma profiles revealed that protonated caffeine possesses highly polar regions capable of establishing strong interactions with EG and ${\mathrm{ZnCl}}_{2}$, enhancing the system’s stability. Furthermore, radial distribution functions (RDFs) showed a decrease in the interaction distance between key atoms after incorporating protonated caffeine. The results suggest that this novel DES has promising potential for industrial applications, especially in the extraction of sulfur compounds from fossil fuels due to the activation of the imidazole ring of caffeine. However, further studies are needed to optimize its operating conditions and evaluate its performance on an industrial scale.

## 1. Introduction

The synthesis of alternative solvents has emerged as a necessary response to the growing environmental challenges posed by the extensive use of conventional solvents in various industrial processes. These processes involve the use of large amounts of organic solvents for chemical reactions, extraction, separation, and purification, among other applications [1]. Although these solvents play a crucial role in chemical processes by improving solubility, dissolution capacity, and chemical stability, many are highly toxic and have adverse effects on both the environment and human health [2].

One promising alternative to conventional solvents is ionic liquids (ILs), which are defined as salts that remain in a liquid state at temperatures below 100 °C, with many remaining liquids at room temperature. ILs consist of an organic cation and an organic or inorganic anion, forming chemically stable species due to strong electrostatic interactions between ions [3]. This unique structure imparts ILs with distinctive properties, such as low vapor pressure, chemical and thermal stability, and the ability to dissolve both organic and inorganic compounds [4].

Despite the advantages of ILs, their synthesis can be complex and often involves costly components, prompting interest in deep eutectic solvents (DESs) as a more accessible alternative. DESs are mixtures of two or more chemical species, typically referred to as hydrogen bond acceptors (HBAs) and hydrogen bond donors (HBDs). When combined in specific molar ratios, these mixtures undergo a significant reduction in their melting points, becoming liquid at temperatures below 100 °C [5]. The components commonly used for DES synthesis are generally safe, inexpensive, and readily available, making them attractive for various industrial applications [6]. A common example involves quaternary ammonium salts, such as choline chloride, as HBAs, while urea, alcohols, amides, amines, or carboxylic acids serve as HBDs [7,8]. The precise combination of HBAs and HBDs directly influences the physicochemical properties of DESs, enabling the tailoring of their applications. In particular, the use of metal salts as HBAs and alcohols as HBDs has been proven advantageous due to their ability to form stable structures through hydrogen bonding and metal coordination [9].

Since 2001 [10], efforts have been made to develop deep eutectic solvents (DESs) as alternative sustainable solvents, particularly in extraction and separation processes. The versatility of DESs has allowed their application in various fields, including catalysis, electrochemistry, and biomass processing. One of their most studied applications is in extractive and oxidative desulfurization processes, which aim to overcome the limitations of conventional hydrodesulfurization (HDS). These limitations include extreme temperature and pressure conditions, high energy and hydrogen consumption, high catalyst costs, and low efficiency in removing aromatic sulfur compounds such as benzothiophene (BT), dibenzothiophene (DBT), and their alkyl derivatives [11,12,13].

DESs offer a promising approach to fuel desulfurization, supporting stricter environmental regulations that limit sulfur content in fuels to 10 ppm in Europe, China, and the United States [14,15]. Various DESs have been synthesized and evaluated for their sulfur extraction efficiency. For example, Gano et al. [16] demonstrated that FeCl_3_-based DESs exhibit over 90% DBT solubility, outperforming ZnCl_2_-based DESs. Further studies optimized DES compositions, achieving sulfur removal efficiencies of up to 99.5% in some cases [17,18,19].

Given these advancements, this study proposes the incorporation of caffeine into DES synthesis due to its unique chemical structure. Caffeine contains a pyrimidinedione and an imidazole ring, making it a versatile hydrogen bond donor and acceptor. Its widespread use in organic synthesis stems from its low toxicity, ease of handling, and lower cost compared to imidazole, which was priced at 130 USD/kg in January 2025 [20].

Although IL-based extractions have shown promising results, DESs have gained attention for their potential in sulfur removal from fossil fuels. In 2013, Li et al. [21] synthesized DESs using choline chloride, tetramethylammonium chloride, and tetrabutylammonium chloride as HBAs, combined with various alcohols and acids as HBDs. A TBAC/polyethylene glycol DES achieved 82.83% BT removal in a single cycle and up to 99.48% after five cycles.

This study explores, for the first time, the incorporation of protonated caffeine into DES synthesis. Its heterocyclic structure enables strong hydrogen bonding interactions, stabilizing both polar and nonpolar compounds [22]. In its protonated form, caffeine enhances electrostatic interactions within the DES, improving molecular stability. Additionally, its dual role as a hydrogen bond donor and acceptor expands the potential applications of DESs. While DESs have proven effective in extractive desulfurization, integrating caffeine opens new possibilities in separation and catalytic processes, with potential applications in fuel desulfurization pending further optimization and evaluation.

## 2. Results and Discussion

### 2.1. Activation of Caffeine’s Imidazole Ring

**Figure 1:**
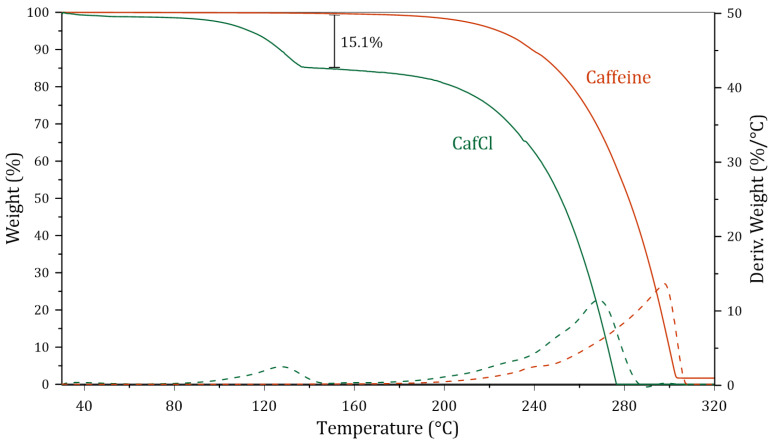
Thermogravimetric analysis of pure and protonated caffeine.

Figure 1 presents the thermogravimetric analysis results for pure caffeine and protonated caffeine. In the case of protonated caffeine, a thermal degradation event is observed between 70 and 75 °C, corresponding to the release of hydrogen chloride from the caffeine molecule. This process accounts for a weight loss of 15.1%. Comparing this value with the theoretical weight percentage of HCl in the CafCl molecule (15.8%), a relative error of 4.43% is calculated, indicating a purity of 95.57% for the protonated CafCl.

**Figure 2:**
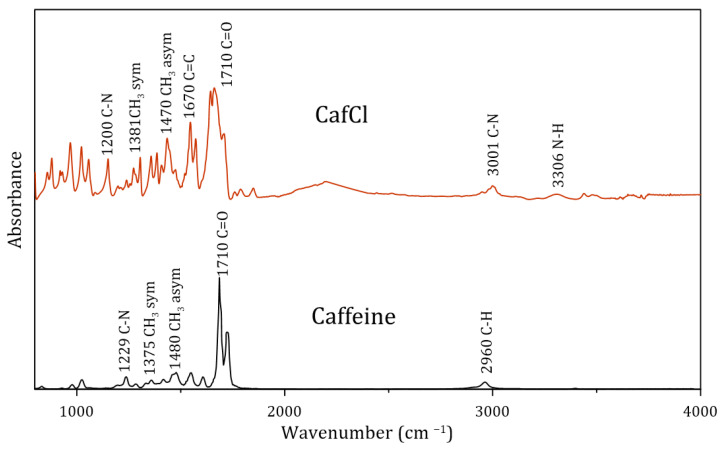
Comparative FTIR spectra of pure caffeine and protonated caffeine (CafCl).

In contrast, the thermogravimetric analysis of pure caffeine reveals a single thermal event occurring at approximately 170 °C, which is attributed to sublimation. Under these conditions, solid caffeine transitions directly into vapor without passing through a liquid phase, as reported in previous studies [23,24]. Once the absence of solvent in the protonated caffeine was confirmed (TGA analysis), the FTIR spectrum was taken as observed in Figure 2.

The presence of the vibrational mode of the N-H bond was identified, which, according to Salami and Ezabadi, occurs at 3306 cm^−1^ [25]. This vibrational mode corresponds to the symmetric stretching of the N-H bond, consistent with the experimental spectrum presented in Figure 2. Furthermore, this observation aligns with Wade’s description [26], which indicates that the N-H stretching frequency should appear as a broad and moderately intense band, near 3300 cm^−1^.

In contrast, the spectrum of pure caffeine revealed the absence of bands corresponding to amino groups (N-H). Instead, molecular vibrations characteristic of other functional groups were observed, including the following: C=O at 1700 cm^−1^, C=C at 1640 cm^−1^, asymmetric bending of ${\mathrm{CH}}_{3}$ at 1462 cm^−1^, symmetric bending of ${\mathrm{CH}}_{3}$ at 1380 cm^−1^, C-H at 3050 cm^−1^, and C-N at 1200 cm^−1^ [27]. These bands are commonly found in both pure caffeine and caffeine hydrochloride (CafCl).

**Figure 3:**
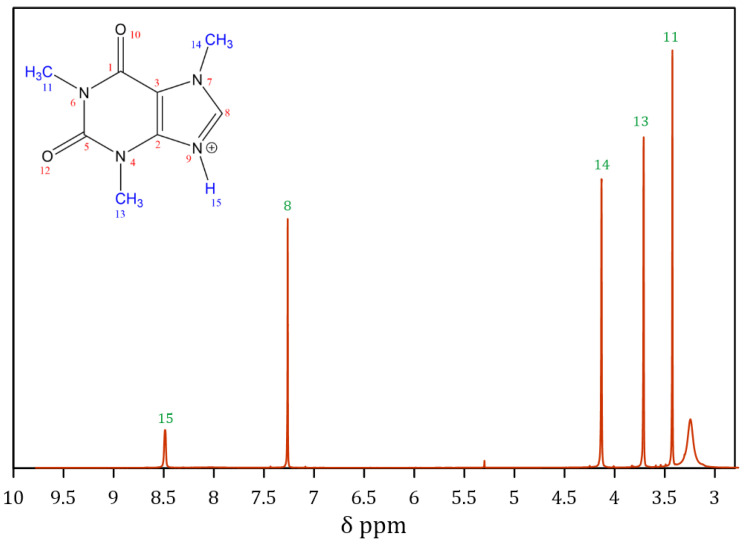
NMR spectrum of protonated caffeine (CafCl).

Additionally, the presence of protonated caffeine was confirmed through Proton Nuclear Magnetic Resonance (^1^H NMR) analysis. The ^1^H NMR spectrum provided insights into the interactions between hydrogen atoms and their neighboring atoms within the molecule. These interactions were expressed as distinct chemical shifts, allowing the identification of different chemical environments of hydrogen atoms in the sample [28]. The ^1^H NMR spectrum of CafCl is shown in Figure 3.

From the spectrum, five significant peaks corresponding to the five protons present in the CafCl molecule can be observed. These peaks were compared with values reported in the literature to determine the chemical shift (δ) and resonance frequencies for each proton group in the molecule, as summarized in Table 1.

The δ values are influenced by the electronic environment surrounding the nucleus, which can be affected by factors such as the presence of functional groups, multiple bonds, and electronegative atoms. In pure caffeine, the methyl groups (${\mathrm{CH}}_{3}$) typically appear as singlet peaks in the NMR spectrum, with chemical shifts ranging from 2.8 to 4 ppm. This is consistent with the observations in Figure 3 for protons 11, 13, and 14 [29]. Proton 8, located in the imidazole ring, typically resonates in the lower chemical shift region (approximately 7–8 ppm). This shift is attributed to electronic shielding caused by electronegative atoms and π-electron delocalization in the aromatic system. In Figure 3, proton 8 is observed at 7.25 ppm, in agreement with literature values [29].

An additional peak, corresponding to proton 15, is present in the CafCl spectrum and represents the proton attached to the nitrogen atom of the imidazole ring, introduced through caffeine protonation. This proton is absent in the spectrum of pure caffeine. According to Salami and Ezabadi [25], this proton resonates at 11.27 ppm; however, their analysis was conducted in dimethyl sulfoxide (DMSO) as the solvent. The solvent’s properties significantly influence the chemical shift, as interactions between the sample and the solvent can alter the electronic distribution. In the case of CafCl, this proton is expected to experience a slight shift towards lower values (higher ppm) compared to free amino groups. This is attributed to additional delocalization of electron density, likely caused by hydrogen bond formation [30].

### 2.2. Synthesis of Protonated Caffeine-Based DES

The formation of the binary DES (${\mathrm{ETG:ZnCl}}_{2}$) is primarily attributed to the ability of ETG to donate hydrogen bonds via its two hydroxyl groups, enabling the formation of hydrogen bonds with the chloride anions (${\mathrm{Cl}}^{-}$) of ${\mathrm{ZnCl}}_{2}$ [31]. Additionally, the possibility of coordination complexes forming between ${\mathrm{Zn}}^{2+}$ ions and the hydroxyl groups of ETG is significant, as ${\mathrm{Zn}}^{2+}$, acting as a Lewis acid, exhibits a strong affinity for the oxygen atoms in the hydroxyl groups [32].

**Figure 4:**
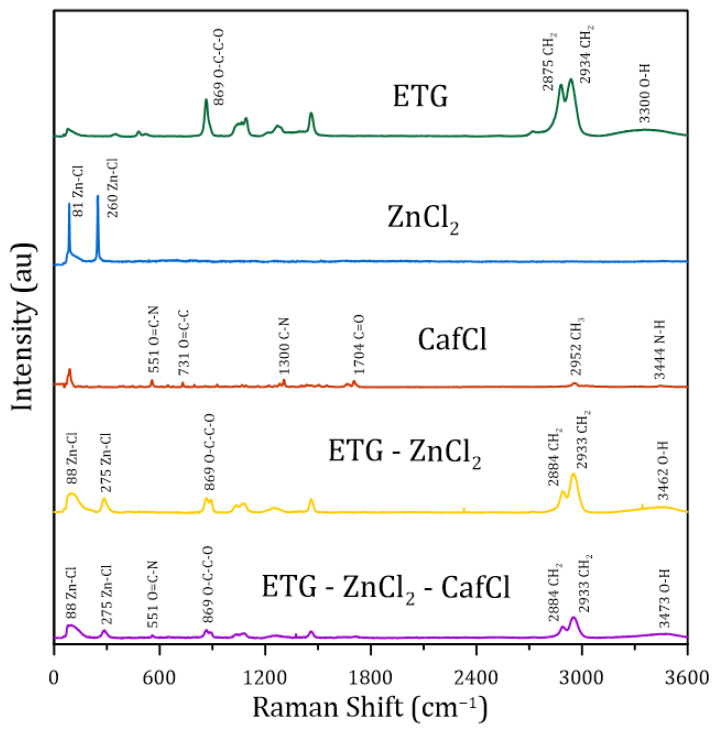
Raman spectra of pure compounds and synthesized DES.

The system’s capacity to incorporate CafCl can be explained by the formation of hydrogen bonds between the carbonyl groups and nitrogen atoms of CafCl with the hydroxyl groups of ETG. Furthermore, it is plausible that stable coordination complexes are formed between ${\mathrm{Zn}}^{2+}$ ions and the nitrogen atoms of CafCl, further enhancing the solubility of CafCl in the DES [33,34]. To better understand these interactions, Raman spectroscopy was used to characterize the pure components (CafCl, ${\mathrm{ZnCl}}_{2}$, ETG), the binary DES (${\mathrm{ETG:ZnCl}}_{2}$), and the ternary DES (${\mathrm{ETG:ZnCl}}_{2}$:CafCl). The corresponding spectra are presented in Figure 4.

For pure CafCl, several significant bands are observed in the Raman spectrum: a vibration at 551 cm^−1^ attributed to the O=C-N deformation of the pyrimidinedione ring [35]; bands at 1659 and 1700 cm^−1^ corresponding to C=O stretching vibrations [36]; a band at 2952 cm^−1^ characteristic of ${\mathrm{CH}}_{3}$ stretching [37]; and a band at 3447 cm^−1^, indicative of N-H vibration, which confirms caffeine protonation [38].

For ${\mathrm{ZnCl}}_{2}$, two prominent bands are observed at 81 cm^−1^ and 281 cm^−1^, the latter being associated with the stretching vibration of the Zn-Cl bond [39]. In the case of EG, the most intense Raman bands appear between 2800 and 3000 cm^−1^, corresponding to ${\mathrm{CH}}_{2}$ stretching, and a broad band between 3300 and 3600 cm^−1^, indicative of OH group vibrations, as reported in the literature [40].

In the binary DES (${\mathrm{ETG:ZnCl}}_{2}$) spectrum, notable similarities with the pure ETG spectrum are observed. Additionally, a band at 282 cm^−1^, which is present in the pure ${\mathrm{ZnCl}}_{2}$ spectrum, is also observed in the DES spectrum. However, this band appears with reduced intensity in the DES, suggesting its involvement in hydrogen bond formation with the OH groups of ETG.

In the ternary DES (${\mathrm{ETG:ZnCl}}_{2}$:CafCl) spectrum, the OH group band shifts to a lower wavenumber (3230 cm^−1^) compared to the binary DES (${\mathrm{EG:ZnCl}}_{2}$) spectrum, where it appears at 3310 cm^−1^. Additionally, an increase in band width is observed, which is a well-documented indicator of hydrogen bond formation, as reported in the literature [41].

Furthermore, the characteristic ${\mathrm{ZnCl}}_{2}$ band at 281 cm^−1^ exhibits a significant decrease in intensity, supporting the hypothesis that ${\mathrm{ZnCl}}_{2}$ participates in hydrogen bond formation with the OH groups of EG. Similarly, the characteristic CafCl band at 551 cm^−1^, attributed to O=C-N deformation, also shows a reduction in intensity, suggesting its involvement in hydrogen bond formation with the OH groups of ETG.

### 2.3. Sigma Profile

**Figure 5:**
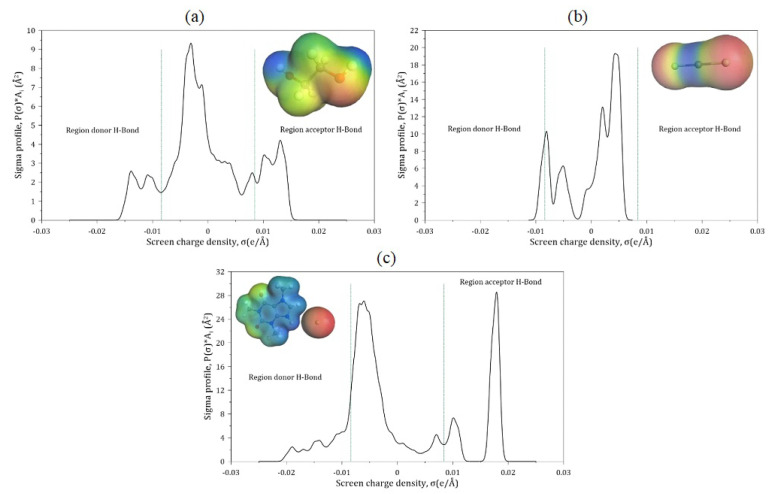
σ-profiles y polarización superficial determinados mediante COSMO-RS para: (**a**) ETG, (**b**) ${\mathrm{ZnCl}}_{2}$ y (**c**) Cl-Caff.

The surface polarization charge density and sigma profile (σ) ETG, ${\mathrm{ZnCl}}_{2}$, and CafCl molecules are depicted in Figure 5 to identify regions prone to hydrogen bond formation and to characterize the distribution of charges on their molecular surfaces. For ETG, its sigma profile spans the range of ±0.016 e/${Å}^{2}$, displaying two prominent peaks. These peaks reveal negative regions localized on the oxygen atoms associated with hydroxyl groups and positive regions on the hydrogen atoms.

Notably, due to the inversion of the polarization charge density sign (σ) relative to the actual polarity of the molecule, the primary peak for oxygen atoms appears on the right side of the profile (approximately +0.0133 e/${Å}^{2}$), while the peak corresponding to hydrogen atoms is located on the left side (around −0.0136 e/${Å}^{2}$). Both peaks surpass the hydrogen bond threshold ($\pm \sigma_{\mathrm{hb}}=\pm$0.0084 e/${Å}^{2}$), and since their magnitudes exceed ±0.01 e/${Å}^{2}$, these regions are classified as strongly polar. Consequently, these areas on ETG’s molecular surface are well suited for forming hydrogen bonds, which aligns with the compound’s high solvation capacity and strong intermolecular interactions. For ${\mathrm{ZnCl}}_{2}$, the sigma profile is predominantly located between −0.01 e/${Å}^{2}$ and +0.006 e/${Å}^{2}$, with prominent peaks reflecting the contributions of the negative charges from the chlorine atoms and the positive charge from the zinc atom. In the σ representation, the peak corresponding to chlorine is found around +0.0045 e/${Å}^{2}$ (right side), while the peak associated with zinc appears near −0.008 e/${Å}^{2}$ (left side). These σ values lie within the range considered nonpolar (±0.0084 e/${Å}^{2}$), indicating a very limited or nearly nonexistent capacity to form strong hydrogen bonds. Consequently, ${\mathrm{ZnCl}}_{2}$ is distinctly different from the ETG molecule in terms of its potential for molecular interactions through hydrogen bonding.

In contrast, the CafCl molecule exhibits a broader sigma profile, spanning approximately ±0.02 e/${Å}^{2}$, with dominant peaks arising from the highly negative regions of the oxygen and chlorine atoms and the highly positive regions of the hydrogen atoms. Due to the inversion of the polarization charge density (σ) sign relative to the actual polarity, the peaks for oxygen and chlorine are located on the right side of the profile (approximately +0.0104 e/${Å}^{2}$ for oxygen and +0.018 e/${Å}^{2}$ for chlorine), while the peaks for hydrogen appear on the left side (near −0.019 e/${Å}^{2}$, −0.0139 e/${Å}^{2}$, and −0.0107 e/${Å}^{2}$). These values significantly exceed the hydrogen bond threshold of ±0.0084 e/${Å}^{2}$, indicating that the CafCl molecule has several highly polar regions (beyond ±0.01 e/${Å}^{2}$) with strong potential to form hydrogen bonds. Thus, CafCl exhibits behavior similar to ETG in its capacity to establish intermolecular interactions through hydrogen bonding.

### 2.4. Radial Distribution Functions

To further investigate the interactions and structure of the DES under study, an analysis of the radial distribution function (RDF) for atom–atom interactions was performed for both the eutectic mixture ${\mathrm{ETG:ZnCl}}_{2}$ and the eutectic mixture ${\mathrm{ETG:ZnCl}}_{2}$:CafCl. Understanding the predominant interactions is essential to elucidate the behavior of this system. The RDF describes the probability of finding a particle at a certain distance from another and can be determined using the following Equation (1) [42]:

(1) $$g\left(r\right)=\frac{\Delta N_{i,j}\left(r,r+\Delta r\right)\cdot V}{4\pi r^{2}\Delta rN_{i}N_{j}}$$

where $N_{i}$ and $N_{j}$ represent the number of particles *i* and *j*, and *V* is the particle volume. Furthermore, *r* is the distance between particles *i* and *j*, and $\Delta N_{i,j}$ indicates the average number of particles *j* at a distance *r* to r+Δr from particle *i*.

**Figure 6:**
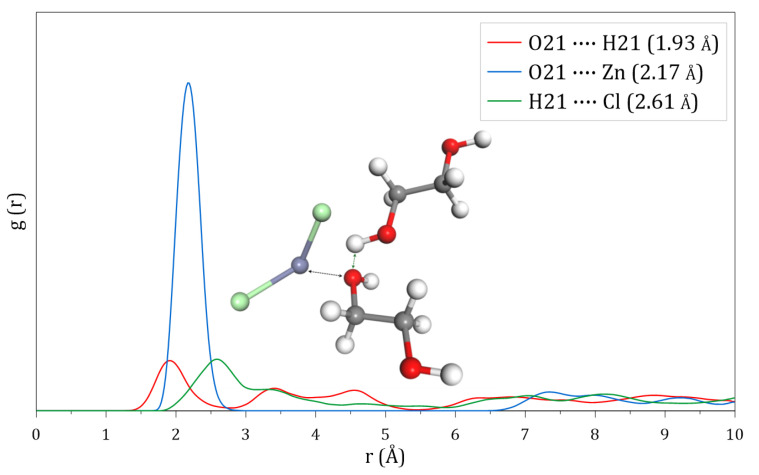
Radial distribution functions calculated at 50 °C for the binary DES composed of ETG and ZnCl_2_.

The analysis of the sigma profile between ETG molecules shows that oxygen atoms act as hydrogen bond acceptors, while hydrogen atoms can serve as donors. Consequently, the radial distribution function between ETG molecules exhibits a prominent peak at 1.93 Å, indicating a high probability of interaction between the hydrogen and oxygen atoms of different ETG molecules, as shown in Figure 6.

Regarding the interactions between ETG and ZnCl_2_, the results show that Zn atoms have a higher probability of interacting with the oxygen atoms of ETG at 2.17 Å, while chlorine atoms associate with the hydrogen atoms of ETG at 2.61 Å. Based on the sigma profile of ZnCl_2_, it is reasonable to infer the existence of strong O–Zn interactions between ETG and ZnCl_2_, while the interactions between chlorine and hydrogen atoms would be considerably weaker.

Upon incorporating the CafCl molecule into the binary DES system, a reduction in the interaction distance of the RDF) between ETG molecules is observed, reaching 1.77 for O-H pairs. Similarly, the RDF between ETG and ${\mathrm{ZnCl}}_{2}$ decreases to 2.11 Å, for interactions between the O-Zn atoms of both molecules. Likewise, the interaction distance between H-Cl atoms is reduced to 2.17.

**Figure 7:**
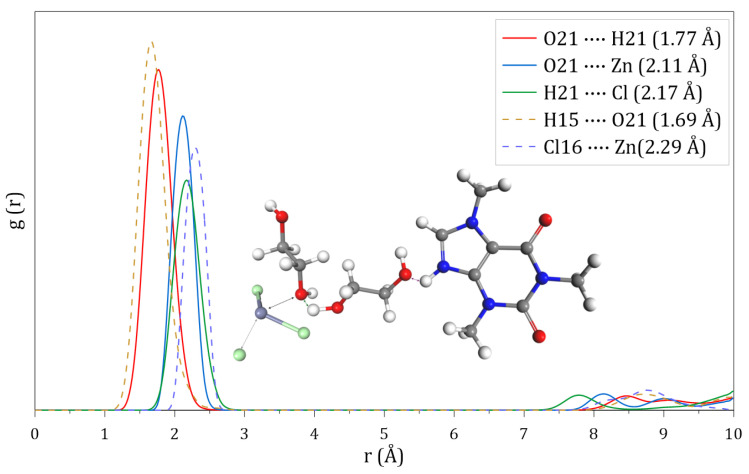
Radial distribution functions calculated at 50 °C for the tertiary DES composed of ETG, ZnCl_2_ and CafCl.

A noteworthy aspect is the predominant interaction between the CafCl molecule and the binary DES. The RDF reveals that the hydrogen atom attached to the nitrogen of the imidazole ring exhibits a strong interaction with the oxygen of ETG, at a distance of 1.69 Å, making it the most significant interaction within the ternary DES. Additionally, a notable interaction is identified between the chloride ion of CafCl and the Zn of ${\mathrm{ZnCl}}_{2}$, with a distance of 2.29. These interactions are illustrated in Figure 7.

The expected behavior of the ternary DES can be approximated based on binary systems composed of ethylene glycol and zinc chloride, for which viscosity, density, melting point, and ionic conductivity values are well documented [43,44]. The incorporation of caffeine into these binary systems may modify these properties by influencing the hydrogen bonding network and ionic mobility. The system’s density could slightly decrease, as caffeine is an organic compound with a lower density than metal halides. Regarding viscosity, an increase is likely due to the formation of additional interactions between protonated caffeine and other components, which restrict molecular motion. The melting point may rise since the presence of organic salts in DESs generally enhances thermal stability. Finally, ionic conductivity may vary depending on caffeine’s effect on the dissociation of charged species and the mobility of ions in the mixture.

## 3. Materials and Methods

### 3.1. Activation of Caffeine’s Imidazole Ring

#### 3.1.1. Chemical Reagents

Commercial caffeine (Sigma-Aldrich, St. Louis, MO, USA 99%) was dried in an oven at 80 °C for 24 h to remove any residual moisture prior to use. Ethanol for synthesis (Sigma-Aldrich, 99%) was treated with zeolites to minimize water content and ensure the solvent was as dry as possible. Sodium chloride (Merk, Darmstadt, Germany 99.5%) was similarly dried at 80 °C for 24 h before use. Sulfuric acid (Aldrich, St. Louis, MO, USA, 95–97%) was used without further purification. It is important to emphasize that all reagents were of analytical grade, and stringent precautions were taken to avoid contamination with moisture.

#### 3.1.2. Reaction Setup and Procedure

The protonation of caffeine at one of the nitrogen atoms in the imidazole ring was performed following the methodology described by Salami and Ezabadi [25], in which caffeine reacts with 37% hydrochloric acid in an aqueous medium at 70 °C for 48 h. To optimize the reaction conditions and ensure the desired product, several modifications were introduced. One of the key improvements was the removal of water from the system, which reduced the reaction time to just 3 h. This was achieved by leveraging the high solubility of gaseous hydrogen chloride in anhydrous ethanol, significantly enhancing the efficiency of the process.

**Figure 8:**
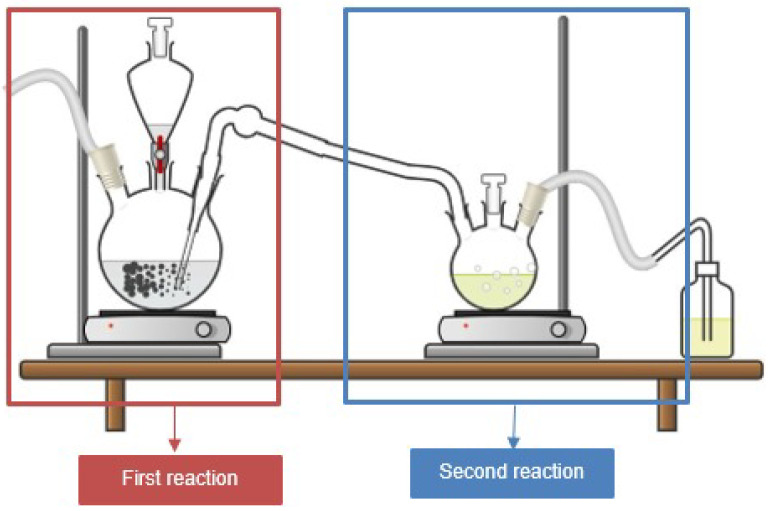
Setup for caffeine protonation.

Furthermore, the presence of water can affect protonation, as it acts as a weak base and competes with caffeine for protons. An excess of water may capture the available protons, thereby reducing the effectiveness of the process. To mitigate this effect, the reaction was conducted in two distinct stages, as illustrated in Figure 8.

**Figure 9:**
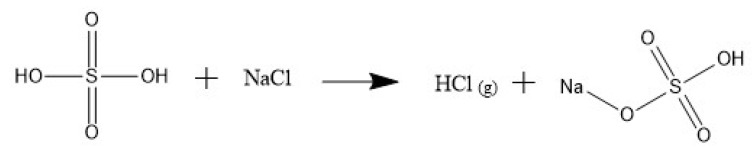
Reaction for production of gaseous hydrogen chloride.

**Figure 10:**
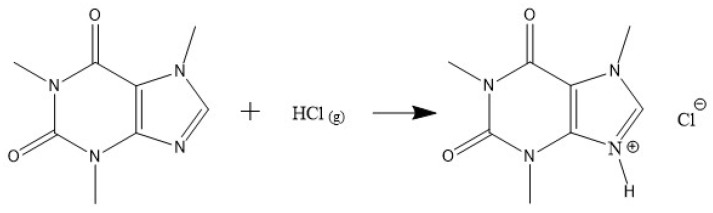
Caffeine protonation reaction.

- First stage, production of anhydrous hydrogen chloride: Anhydrous hydrogen chloride was generated by the reaction between sodium chloride and sulfuric acid, as shown in Figure 9. The system consisted of a round-bottomed flask containing sodium chloride, with sulfuric acid added dropwise, while the mixture was maintained at a constant temperature of 150 °C using a heating mantle, as reported by Groover [45]. An excess of hydrogen chloride (5 molar equivalents) was bubbled into the ethanol solution containing caffeine to drive the equilibrium of the protonation reaction toward product formation, ensuring complete protonation of the caffeine.
- Second stage, protonation reaction: The protonation reaction was carried out at 60 °C (Figure 10) with caffeine dissolved in anhydrous ethanol. A total of 110 mL of anhydrous ethanol was used to fully dissolve the caffeine at this temperature. The gaseous hydrogen chloride produced in the first stage was continuously bubbled into the ethanol solution, leading to the protonation of caffeine, which was indicated by the precipitation of a white solid after 1 h of reaction.
- Purification of caffeine hydrochloride: The solid product, caffeine hydrochloride (CafCl), was purified through rotary evaporation at 55 °C and 200 mbar to eliminate excess ethanol. The resulting solid was subsequently dried in a vacuum oven at 55 °C for 24 h to ensure complete removal of residual solvents. The final product yield was 88.4%.

#### 3.1.3. Characterization of Protonated Caffeine (CafCl)

The synthesized product, identified as caffeine hydrochloride (CafCl), was characterized using nuclear magnetic resonance spectroscopy (¹H NMR), Fourier-transform infrared spectroscopy (FTIR), and thermogravimetric analysis (TGA). The ¹H NMR spectrum was acquired using a Bruker Ascend III HD 600 MHz high-performance spectrometer equipped with a Prodigy TCI 5 mm cold probe, with deuterated chloroform as the solvent (Bruker, Ettlingen, Germany). The spectral data were processed with M-Nova 14.2 software, including baseline correction applied to the raw signals.

For FTIR analysis, the sample was prepared using standard KBr pellet techniques and analyzed over the spectral range of 4000–800 cm^−1^ using a Thermo Fisher Scientific Nicolet 6700 spectrometer equipped with an MCT detector (Thermo Fisher Scientific, Waltham, MA, USA). The spectra were baseline-corrected to zero absorbance, smoothed, and normalized to the maximum intensity signal to facilitate comparisons with reference databases and the existing literature.

Thermogravimetric analysis (TGA) was performed on a TA Instruments Q500 instrument (TA Instruments, New Castle, DE, USA). The analysis focused on determining weight loss and maximum decomposition temperature, with the raw data processed accordingly to provide detailed thermal stability profiles.

### 3.2. Synthesis of Protonated Caffeine-Based DES

#### 3.2.1. Chemical Reagents

The previously synthesized caffeine chloride (CafCl) was used along with the following chemical reagents: choline chloride (99%), zinc chloride (99%), urea (99%), and citric acid (99.5%) from Sigma-Aldrich; tetraethylammonium bromide (98%), ethylene glycol (99%), glycerol (98%), thiourea (99%), and malonic acid (99%) from Merck; tetraethylammonium chloride (99%), succinic acid (99%), acetamide (99%), N-methylurea (97%), adipic acid (99%), benzoic acid (99%), and benzamide (99%) from Fisher Scientific (Waltham, MA, USA).

Since the synthesis of deep eutectic solvents (DESs) using protonated caffeine has not been reported in the literature, different combinations of HBAs and HBDs were tested until the incorporation of protonated caffeine into the system was achieved, resulting in a liquid with manageable viscosity at temperatures below 100 °C. The HBAs and HBDs used in the synthesis are detailed in Table 2.

DES synthesis was performed in a 50 mL beaker, maintaining the mixture at 50 °C with constant stirring at 200 rpm to ensure the incorporation of CafCl and prevent its degradation. Initially, the HBAs and HBDs were mixed to form the eutectic mixture. Once a decrease in the melting point of the mixture was observed, increments of 0.05 g of protonated caffeine were added to determine the maximum amount that the system could incorporate. The synthesized DESs are presented in Table 2.

As shown in Table 2, the DES that demonstrated successful incorporation of CafCl was composed of ETG and ZnCl_2_ in a molar ratio of 2:1. Up to 0.5 g of CafCl was incorporated into the system, resulting in a ternary DES with a molar ratio of ETG:ZnCl_2_:CafCl of 2:1:0.1.

#### 3.2.2. Characterization of the Synthetized DES

To verify the structure of the binary DES (ETG:ZnCl_2_) and the ternary DES (ETG:ZnCl_2_:CafCl), characterizations were performed using Raman spectroscopy with a Labram HR model instrument. This technique enabled the confirmation of hydrogen bond formation between hydrogen bond acceptors and hydrogen bond donors. Additionally, the pure compounds used (ETG and ZnCl_2_) were also characterized. The obtained spectra were analyzed using SpectraGryph 1.2 software, with baseline corrections applied to ensure the accuracy of the results.

### 3.3. Computational Detail

#### 3.3.1. COSMO-RS Calculation

The conductor-like screening model for realistic solvents (COSMO-RS) was used to calculate the thermodynamic properties of the components of the ternary DES (ETG:ZnCl_2_:CafCl). Quantum-based equilibrium thermodynamic methods provide a robust framework to describe the molecular behavior of DES components, demonstrating good qualitative and quantitative predictive capabilities [46]. The central principle of COSMO-RS involves the creation of a virtual conductor, which simulates the introduction of molecules into an aqueous medium through continuous solvation models to determine their charge distribution [47].

Using charge distribution in the form of sigma profiles, which describe the polarity of molecules, COSMO-RS qualitatively identified regions where hydrogen bonding occurs. In sigma profile graphs, each peak represents the charge density of an atom within the molecule. Atoms with a positive partial charge appear as negative charge densities, and vice versa [47]. Sigma profiles represent surface charge densities rather than direct partial charges. This results in an inversion of sign because the molecular cavity is embedded in a virtual conductor, where the screening charge on the cavity surface compensates for the molecular charge distribution. Consequently, a positively charged region within the molecule induces a negative screening charge on the surface, and a negatively charged region induces a positive screening charge, leading to the observed sign inversion in sigma profiles [48].

To obtain sigma profiles, the geometry of each DES component was created and optimized using the density functional theory (DFT) electronic structure program DMol^3^ [49,50]. Graphical visualizations were generated with Materials Studio 2017 (Accelrys Software Inc., San Diego, CA, USA). Once the structure of each component was optimized, the Becke–Perdew [51] version of the Vosko–Wilk–Nusair functional [52] was applied to achieve a more accurate characterization of the charge distribution [53]. Following the calculations, the sigma profiles of each DES component were generated.

#### 3.3.2. Ab Initio Molecular Dynamics (AIMD)

**Figure 11:**
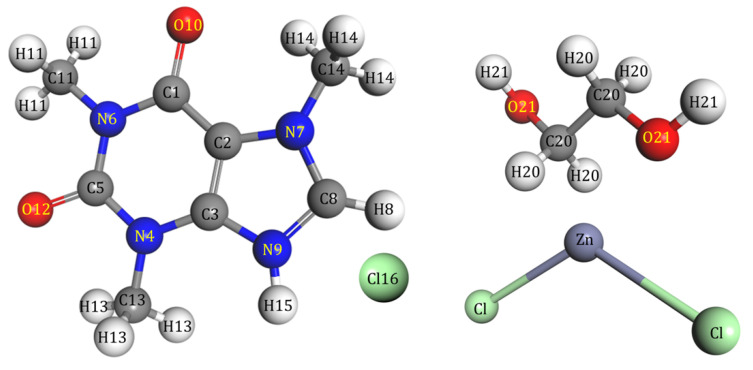
Schematic representation of ethylene glycol (ETG), zinc chloride (${\mathrm{ZnCl}}_{2}$), and protonated caffeine (CafCl).

In this study, ab initio molecular dynamics (AIMD) was employed to investigate the structure of the DES composed of ETG:ZnCl_2_:CafCl. Figure 11 illustrates the molecular structure of the components of the DES.

The system under study incorporates a mixture with a molar ratio of ETG:ZnCl_2_:CafCl of 2:1:0,1. This ratio was selected based on experimental findings. A cubic simulation box containing the binary DES composed of 4 ETG molecules and 2 ZnCl_2_ molecules was first generated. Subsequently, one CafCl molecule was added to the previous system to obtain a cubic simulation box ($13\times 13\times 13\;{Å}^{3}$) composed of 4 ETG molecules, 2 ZnCl_2_ molecules, and 1 CafCl molecule.

The AIMD simulations were conducted using density functional theory (DFT) within the DMol^3^ module of the Materials Studio software [54]. The structures of the compounds were fully optimized using DFT with the generalized gradient approximation (GGA) functional and the Becke–Lee–Yang–Parr (BLYP) basis set [55].

To observe and analyze the temporal evolution of the systems over time, AIMD simulations were performed under periodic boundary conditions to avoid boundary effects. The simulation boxes for both systems were equilibrated for 5.0 ps using a canonical ensemble (NVT) with Nosé–Hoover chain thermostats applied to individual atoms, with a time constant of 100 fs and a timestep of 1.0 fs [56]. The equilibrium temperature was set at 300 K. For the subsequent production stage, a microcanonical ensemble (NVE) was employed, with the temperature set at 323.15 K. The production time was set to 20 ps, while the time constant and timestep remained the same as during the equilibration stage.

## 4. Conclusions

The growing need for more sustainable solvents has led to the development of deep eutectic solvents (DESs) as a viable alternative to conventional solvents, due to their low cost, ease of synthesis, and adjustable properties through the appropriate selection of hydrogen bond donors and acceptors. In this study, the incorporation of protonated caffeine into a DES was explored for the first time, leveraging its chemical structure to enhance the stability and functionality of the system. Caffeine was protonated through an optimized procedure in an anhydrous medium, preventing the presence of water, which allowed for efficient interaction with hydrogen chloride. Characterization via TGA, FTIR, and NMR confirmed the formation of the N-H bond in the imidazole ring, suggesting an increased capacity for interaction with other DES components.

Various combinations of hydrogen bond acceptors and donors were evaluated to determine the optimal composition of the system, revealing that the most stable and functional mixture was obtained with ethylene glycol (HBD) and zinc chloride (HBA) in a molar ratio of 2:1:0.1 (${\mathrm{ETG:ZnCl}}_{2}$:CafCl). Characterization through FTIR and NMR confirmed the formation of hydrogen bonds and the coordinative interaction between the components, verifying the structural stability of the system.

Computational simulations using COSMO-RS and ab initio molecular dynamics (AIMD) enabled the analysis of charge distribution and hydrogen bond formation in the eutectic mixture. The sigma profiles indicated that protonated caffeine possesses highly polar regions capable of establishing strong interactions with ethylene glycol and ${\mathrm{ZnCl}}_{2}$, enhancing the stability of the system. Additionally, radial distribution function (RDF) analysis showed a reduction in the interaction distance between key atoms in the system upon incorporating protonated caffeine, suggesting an improvement in DES stability.

This new DES exhibits great potential for industrial applications, particularly in the extraction of sulfur compounds from fossil fuels, which could contribute to reducing pollutant emissions. The inclusion of protonated caffeine in the eutectic mixture provides additional advantages in terms of thermal stability and solvation capacity.

Although the ternary DES exhibits good thermal stability at 50 °C, its viscosity increases significantly at lower temperatures, which may impact its potential applications. Additionally, long-term stability remains a key concern. As shown in Figure 1, protonated caffeine undergoes thermal degradation at temperatures above 70 °C, potentially affecting both the thermal and chemical stability of the DES under industrial conditions. To overcome these limitations and enhance the industrial applicability of this DES, several strategies can be considered. First, optimizing the synthesis conditions by exploring alternative hydrogen bond donors or acceptors could help reduce viscosity at lower temperatures while maintaining stability. Additionally, incorporating co-solvents or additives may improve fluidity without compromising the DES’s unique properties. To address long-term stability concerns, process modifications such as maintaining operations within an optimal temperature range should be explored. Furthermore, encapsulation techniques or the use of protective atmospheres could minimize moisture absorption and thermal decomposition.

## Figures and Tables

**Table 1:** Proton chemical shift comparison of caffeine and CafCl.

Proton	Theoretical δ Caffeine	Literature δ Caffeine	Experimental δ CafCl	Literature δ CafCl	Experimental Peak Integration
8	7.5	7.58	7.27	7.99	1.0
11	3.48	3.37	3.27	3.13	3.10
13	3.51	3.55	3.71	3.33	3.10
14	4.0	4.01	4.13	3.82	3.04
15			8.5	11.27	0.99

**Table 2:** Observations of HBA and HBD combinations for protonated CafCl incorporation.

HBD	HBA	Molar Ratio	Observations About CafCl Incorporation
Ethylene glycol (ETG)	Choline chloride (ChCl)	2:1	Partial incorporation with high viscosity
Benzoic acid	Choline chloride	1:1	Crystal formation, partial incorporation with high viscosity
Benzamide	Choline chloride	2:1	Partial incorporation with high viscosity
Ethylene glicol	Tetraethylammonium bromide	2:1	Complete incorporation with medium viscosity
Ethylene glicol	Hydrated iron chloride	3:1	Partial incorporation with high viscosity
Ethylene glicol	Zinc chloride	2:1	Complete incorporation with manageable viscosity

## Data Availability

The original contributions presented in this study are included in the article. Further inquiries can be directed to the corresponding author.
